# Whole-Genome Sequences of Staphylococcus aureus Isolates from Cystic Fibrosis Lung Infections

**DOI:** 10.1128/MRA.01564-18

**Published:** 2019-01-17

**Authors:** Eryn E. Bernardy, Robert A. Petit, Abraham G. Moller, Jennifer A. Blumenthal, Alexander J. McAdam, Gregory P. Priebe, Aroon T. Chande, Lavanya Rishishwar, I. King Jordan, Timothy D. Read, Joanna B. Goldberg

**Affiliations:** a Department of Pediatrics, Division of Pulmonology, Allergy/Immunology, Cystic Fibrosis and Sleep, Emory University, Atlanta, Georgia, USA; b Emory-Children’s Center for Cystic Fibrosis Research, Children's Healthcare of Atlanta, Atlanta, Georgia, USA; c Department of Medicine, Division of Infectious Diseases, Emory University, Atlanta, Georgia, USA; d Division of Critical Care Medicine, Department of Anesthesiology, Critical Care and Pain Medicine, Boston Children’s Hospital, Boston, Massachusetts, USA; e Division of Infectious Diseases, Department of Pediatrics, Boston Children’s Hospital, Boston, Massachusetts, USA; f Departments of Anaesthesia and Pediatrics, Harvard Medical School, Boston, Massachusetts, USA; g Department of Laboratory Medicine, Boston Children’s Hospital, Boston, Massachusetts, USA; h Department of Pathology, Harvard Medical School, Boston, Massachusetts, USA; i School of Biological Sciences, Georgia Institute of Technology, Atlanta, Georgia, USA; j IHRC-Georgia Tech Applied Bioinformatics Laboratory, Atlanta, Georgia, USA; k PanAmerican Bioinformatics Institute, Cali, Valle del Cauca, Colombia; Loyola University Chicago

## Abstract

Staphylococcus aureus is an early colonizer in the lungs of individuals with cystic fibrosis (CF), but surprisingly, only a limited number of genomes from CF-associated S. aureus isolates have been sequenced. Here, we present the whole-genome sequences of 65 S. aureus isolates obtained from 50 individuals with CF.

## ANNOUNCEMENT

Cystic fibrosis (CF) is a genetic disease that affects over 70,000 people worldwide. The major cause of death is chronic bacterial lung infections. Early colonization is often with Staphylococcus aureus, and Pseudomonas aeruginosa emerges later as the major cause of mortality. While many researchers focus on P. aeruginosa, the importance of S. aureus in the history of CF lung infections remains understudied (1). Only a small number of whole-genome sequences of CF-associated S. aureus clinical isolates have been reported (1 – 4). We have started to fill that gap by performing whole-genome sequence analysis of 65 CF-associated S. aureus isolates, obtained mostly from sputum samples, after cultivation on blood agar. This collection is composed of 50 isolates (from 36 individuals) from the Emory Cystic Fibrosis Biospecimen Registry (CFBR) and 15 isolates (from 14 individuals) from Boston Children’s Hospital. Metadata associated with the isolates (e.g., methicillin resistance) and the deidentified patient information (e.g., age and sex) were recorded and made available.

DNA was extracted using the Promega Wizard genomic DNA purification kit, and paired-end libraries were constructed for each isolate using the Nextera XT DNA library kit, with a fragment size of 1,000 bp, and sequenced on an Illumina MiSeq platform using version 3 chemistry. One isolate (CFBR_EB_Sa105, BioSample number SAMN09847825), the first sample of six longitudinal samples from a single patient, also underwent long-read genomic DNA sequencing using the Oxford Nanopore MinION sequencer. DNA was extracted in the same manner as before, and sequencing libraries were prepared using the SQK-RAD003 1D rapid sequencing kit and sequenced on a FLO-MIN106 R9.4 flow cell. The raw Illumina reads for each isolate were processed with the Staphopia analysis pipeline (Docker tag 112017, default parameters) (5). Staphopia performed sequence quality control by removing Illumina adapters and low-quality bases and reads (Q < 20). The remaining high-quality reads for each isolate were then subsampled to 100× coverage by Staphopia and assembled using the *de novo* assembler SPAdes (version 3.11.1, default parameters) (6). Illumina reads and Nanopore reads for isolate CFBR_EB_Sa105 were assembled using the hybrid assembler Unicycler (version 0.4.0, default parameters) (7).

CFBR_EB_Sa105 was sequenced to a total 397× genome coverage (Nanopore, 250×; Illumina, 147×) and assembled into a single 2,782,740-bp-long contig. Genome coverage for the remaining 64 isolates with only Illumina sequencing ranged from 111× to 395×, with an average of 192× coverage, which is sufficient to produce a reliable draft assembly. The *N*_50_ values ranged from 122,702 bp to 806,706 bp, with an average *N*_50_ value of 370,074 bp. The assembled genome sizes ranged from 2,656,854 bp to 2,967,979 bp, with an average size of 2,799,404 bp. The GC contents for these assemblies ranged from 32.59% to 32.85%, with an average GC content of 32.74%. The genome size and GC content values are consistent with what is expected for S. aureus. All assemblies were annotated with the Prokaryotic Genome Annotation Pipeline available from the National Center for Biotechnology Information (NCBI) (8, 9).

Genome sequence analysis along with the metadata of these S. aureus isolates can be used to understand phenotypic adaptations of this pathogen that are required for survival within the multispecies community of the CF lung.

### Data availability.

The complete assembly for CFBR_EB_Sa105 and draft assemblies for the remaining samples, as well as the raw Illumina reads, have been deposited in NCBI and are available under BioProject accession number PRJNA480016. Information for each isolate, including accession numbers, is shown in Table 1.

**TABLE 1:** Sequencing and assembly metrics for *Staphylococcus aureus* CF lung infection isolates in this study

Sample name	SRA accession no.	NCBI RefSeq or GenBank assembly accession no.	Genome coverage (×)	Total length (bp)	No. of contigs	*N*_50_ length (bp)	GC content (%)
CFBR_EB_Sa105	SRX4563672, SRX4596105	NZ_CP031779	147; 250	2,782,740	1	2,782,740	32.78
BCH-SA-01	SRX4563649	RIWH00000000	168	2,788,868	43	266,842	32.77
BCH-SA-02	SRX4563648	RIWI00000000	173	2,791,081	31	443,670	32.77
BCH-SA-03	SRX4563647	RIWJ00000000	177	2,695,371	47	443,662	32.71
BCH-SA-04	SRX4563646	RIWK00000000	220	2,741,344	31	711,701	32.72
BCH-SA-05	SRX4563645	RIWL00000000	172	2,702,509	54	137,651	32.76
BCH-SA-06	SRX4563644	RIWM00000000	189	2,743,469	51	244,024	32.78
BCH-SA-07	SRX4563643	RIWN00000000	128	2,734,154	32	672,541	32.71
BCH-SA-08	SRX4563642	RIWO00000000	136	2,871,626	72	150,513	32.74
BCH-SA-09	SRX4563652	RIWP00000000	137	2,738,552	61	242,262	32.85
BCH-SA-10	SRX4563651	RIWQ00000000	287	2,737,369	35	585,329	32.68
BCH-SA-11	SRX4563667	RIWR00000000	153	2,843,748	80	150,770	32.78
BCH-SA-12	SRX4563666	RIWS00000000	179	2,656,854	37	716,196	32.76
BCH-SA-13	SRX4563665	RIWT00000000	113	2,816,350	47	806,706	32.65
BCH-SA-14	SRX4563664	RIWU00000000	111	2,733,025	36	578,911	32.71
BCH-SA-15	SRX4563671	RIWV00000000	268	2,776,591	32	605,345	32.65
CFBR_EB_Sa101	SRX4563670	RIWW00000000	263	2,822,328	67	153,724	32.74
CFBR_EB_Sa102	SRX4563669	RIWX00000000	327	2,819,773	66	136,645	32.72
CFBR_EB_Sa103	SRX4563668	RIWY00000000	295	2,967,979	176	305,135	32.59
CFBR_EB_Sa104	SRX4563673	RIWZ00000000	124	2,818,295	60	153,724	32.73
CFBR_EB_Sa108	SRX4563632	RIXA00000000	156	2,788,410	54	242,088	32.80
CFBR_EB_Sa110	SRX4563633	RIXB00000000	124	2,787,015	49	222,157	32.79
CFBR_EB_Sa112	SRX4563630	RIXC00000000	261	2,787,692	49	222,157	32.80
CFBR_EB_Sa114	SRX4563631	RIXD00000000	172	2,787,023	49	242,088	32.78
CFBR_EB_Sa116	SRX4563628	RIXE00000000	303	2,851,502	61	441,461	32.75
CFBR_EB_Sa117	SRX4563629	RIXF00000000	134	2,723,586	49	430,410	32.75
CFBR_EB_Sa118	SRX4563626	RIXG00000000	155	2,811,738	49	441,461	32.80
CFBR_EB_Sa119	SRX4563627	RIXH00000000	146	2,840,258	80	158,059	32.66
CFBR_EB_Sa121	SRX4563637	RIXI00000000	152	2,861,087	47	378,754	32.62
CFBR_EB_Sa122	SRX4563638	RIXJ00000000	130	2,866,137	148	142,136	32.72
CFBR_EB_Sa123	SRX4563641	RIXK00000000	133	2,748,775	50	222,157	32.76
CFBR_EB_Sa125	SRX4563640	RIXL00000000	264	2,902,592	68	209,697	32.70
CFBR_EB_Sa126	SRX4563650	RIXM00000000	111	2,793,970	44	603,205	32.63
CFBR_EB_Sa127	SRX4563661	RIXN00000000	126	2,936,734	68	345,554	32.65
CFBR_EB_Sa129	SRX4563677	RIXO00000000	166	2,881,105	38	403,760	32.64
CFBR_EB_Sa130	SRX4563634	RIXP00000000	148	2,879,733	41	605,839	32.63
CFBR_EB_Sa131	SRX4563639	RIXQ00000000	136	2,769,595	42	393,217	32.84
CFBR_EB_Sa133	SRX4563636	RIXR00000000	111	2,880,683	40	605,719	32.63
CFBR_EB_Sa135	SRX4563675	RIXS00000000	149	2,809,235	45	380,091	32.66
CFBR_EB_Sa138	SRX4563674	RIXT00000000	279	2,813,106	35	592,726	32.68
CFBRSa03	SRX4563678	RIXU00000000	326	2,714,903	43	206,745	32.75
CFBRSa04	SRX4563679	RIXV00000000	272	2,790,920	49	161,318	32.77
CFBRSa05	SRX4563680	RIXW00000000	147	2,791,679	37	574,969	32.63
CFBRSa06	SRX4563681	RIXX00000000	122	2,898,057	83	174,877	32.65
CFBRSa07	SRX4563682	RIXY00000000	320	2,817,443	62	351,360	32.85
CFBRSa21	SRX4563683	RIXZ00000000	170	2,784,304	37	434,050	32.70
CFBRSa22	SRX4563684	RIYA00000000	111	2,927,132	117	221,278	32.82
CFBRSa23	SRX4563685	RIYB00000000	138	2,748,954	42	443,595	32.77
CFBRSa24	SRX4563686	RIYC00000000	119	2,790,775	43	443,500	32.77
CFBRSa25	SRX4563687	RIYD00000000	167	2,828,883	46	441,089	32.79
CFBRSa26	SRX4563660	RIYE00000000	243	2,822,821	71	136,638	32.74
CFBRSa27	SRX4563659	RIYF00000000	113	2,751,330	62	122,702	32.77
CFBRSa28	SRX4563658	RIYG00000000	303	2,761,455	33	587,976	32.78
CFBRSa29	SRX4563657	RIYH00000000	258	2,926,156	52	379,601	32.66
CFBRSa30	SRX4563656	RIYI00000000	140	2,741,496	40	288,439	32.76
CFBRSa47	SRX4563655	RIYJ00000000	123	2,787,985	49	242,088	32.79
CFBRSa48	SRX4563654	RIYK00000000	138	2,702,434	39	304,792	32.76
CFBRSa49	SRX4563653	RIYL00000000	147	2,895,019	46	302,780	32.64
CFBRSa50	SRX4563663	RIYM00000000	313	2,753,672	37	500,538	32.81
CFBRSa51	SRX4563662	RIYN00000000	267	2,662,831	40	262,319	32.76
CFBRSa66A	SRX4563688	RIYO00000000	395	2,783,493	37	443,669	32.80
CFBRSa66B	SRX4563689	RIYP00000000	114	2,782,586	35	443,669	32.80
CFBRSa70	SRX4563676	RIYQ00000000	244	2,691,630	42	500,528	32.72
CFBRSa74	SRX4563635	RIYR00000000	298	2,823,147	43	321,988	32.81
JE2	SRX4563690	RIYS00000000	319	2,863,521	66	606,166	32.76
